# Indocyanine green and height of anastomosis in colorectal surgery– a network meta-analysis

**DOI:** 10.1007/s00423-025-03765-x

**Published:** 2025-06-12

**Authors:** Kar Yin Fok, James Wei-Tatt Toh

**Affiliations:** 1 Department of Colorectal Surgery, Westmead Hospital, Cnr Hawkesbury and Darcy Rds, Westmead, NSW 2047 Australia; 2 University of Sydney, Camperdown, NSW 2050 Australia

**Keywords:** Indocyanine green, Anastomotic leak, Colorectal anastomosis, High anterior resection, Low anterior resection

## Abstract

**Purpose:**

Anastomotic leak is a potentially life-threatening complication of colorectal surgery, with perfusion and height of anastomoses considered important risk factors. Indocyanine green (ICG) is commonly used in fluorescence angiography (FA) for perfusion assessment though techniques vary. This is a network meta-analysis comparing use of ICG-FA and height of anastomosis for left sided colorectal anastomoses and rates of anastomotic leak.

**Methods:**

A systematic review was performed including all adult clinical studies using ICG-FA in colorectal anastomoses. A network meta-analysis was performed to compare high and low anterior resections and the use of ICG for the outcome of anastomotic leak.

**Results:**

Of some 333 studies retrieved during review, 31 studies were included, totalling 6431 patients. In a meta-analysis to compare high and low anterior resection, with and without ICG, odds of anastomotic leak were greater in low compared to high anastomoses, and ICG is protective in both, OR of 0.38 (0.20–0.73) for high and OR of 0.41 (0.30–0.54) in low anastomoses, with ICG compared to without.

**Conclusion:**

There is benefit of ICG for both HAR and LAR in reducing anastomotic leak. While there is no consensus on the application, dosage and timing of ICG during anterior resection, pooled results and most studies have shown that the use of ICG to check for tissue perfusion of anastomosis reduces leak. ICG should be considered as part of a surgeon’s armamentarium for intraoperative anastomotic check to reduce the risk of postoperative anastomotic leak.

**Supplementary Information:**

The online version contains supplementary material available at 10.1007/s00423-025-03765-x.

## Introduction

Anastomotic leak is a serious, potentially life-threatening complication of colorectal surgery, with reported rates of 5–19%, and is associated with increased morbidity and mortality [1]. For colorectal cancer, it may result in worse oncological outcomes, including local recurrence and decreased survival [2]. Its cause is multifactorial, including patient and technical factors. Adequate blood supply to the anastomoses is vital for healing, and inadequate perfusion of anastomosis can lead to anastomotic failure, whether it be leak or stricturing. Assessment of blood flow is traditionally by assessing flow at the marginal arteries prior to ligation, the presence of palpable pulse in the mesentery, assessment of bowel discoloration at the distal margin, and bleeding from resection margin. The issue with this is that for elderly patients, especially with atherosclerotic disease, marginal artery flow may be significantly impaired and it may be difficult to gauge what is adequate flow and what is acceptable for anastomosis.

Indocyanine green (ICG) is a water-soluble, tricarbocyanine dye that binds to plasma proteins, and remains in the intravascular compartment until elimination by biliary excretion. In colorectal surgery, ICG has most widely been studied in perfusion analysis of anastomoses. Fluorescence imaging uses illumination of tissue with light at excitation wavelength, 750–800 nm for ICG, while observing nearby tissue at longer emission wavelengths, greater than 800 nm [3]. It is safe, with low toxicity, and suitable for repeated application.

Fluorescence angiography (FA) allows for visualisation of perfusion intraoperatively in real time. ICG is injected intravenously and is visualised as green when excited by light in the near-infrared (NIR) spectrum in the tissue of interest. The ICG is seen visually by the surgeon to ensure that the green colour reaches the anastomosis proximally and distally. More objective methods such as the use of software derived fluorescence time curves for assessment have been described, although uncommonly used [4].

Several studies have shown decreased rates of anastomotic leak with ICG-FA, however findings from some recent studies, including randomised trials, have been more conflicting [5, 6]. There is therefore current uncertainty as to when ICG-FA should be used. Some studies have shown use of ICG-FA changed surgical plan for proximity of anastomoses, without changing rates of anastomotic leak [5, 7]. There is, also heterogeneity in technique in the literature, with differences in ICG dosing, timing, visual distance, and platforms used. ICG-FA may be used for perfusion assessment, before, after, or both before and after anastomoses, and some only describe a serosal technique, while others perform an endoscopic mucosal assessment as well.

The height of anastomosis is also important, with lower anastomoses known to be a risk factor for leak [8]. It is a technical and usually non-modifiable risk factor based on the location of pathology, be it benign or malignant. While ICG-FA has been utilised in both right and left sided colorectal anastomoses, to the best of our knowledge, the significance of this in left sided anastomoses has not been separately studied. Given the conflicting results of recent trials on the use of ICG in decreasing the rate of anastomotic leak, it is clinically relevant to know if ICG provides a benefit for high compared to low left-sided colorectal anastomoses, and based on this, whether it should be used selectively or routinely. This study therefore aims to perform a network meta-analysis on the use of ICG-FA and the height of anastomoses, in rates of anastomotic leak.

## Methods

### Database search

The systematic review and network meta-analysis was performed according to PRISMA guidelines and registered with International Prospective Register of Systematic Reviews (PROSPERO; registration number CRD42024500601).

A literature search was performed in electronic databases: MEDLINE, Pubmed, Embase and Cochrane Central Register of Controlled Trials. The following MESH terms were used: colorectal surgery, indocyanine green, fluorescence, anastomosis, surgical, perfusion, perfusion imaging, anastomotic leak, postoperative complications. Additional keywords colon, rectum, ICG*, ICG-FA were used. Boolean operator ‘OR’ for each concept and ‘AND’ to combine concepts. This was supplemented by a manual search through bibliographic references. Databases were searched from inception to January 2024.

## Inclusion and exclusion criteria

All clinical studies with adults over the age of 18 undergoing colorectal surgery with use of ICG for perfusion assessment were included. The search was limited to human studies in the English language; no restriction was set for type of publication.

A network meta-analysis was performed for left sided resections based on the height of anastomosis in conjunction with use of ICG. High anterior resection was defined as having an anastomosis above the peritoneal reflection and low anterior resection when it is below. Where anastomotic height was measured instead, greater than 10 cm from anal verge is defined as high, with low being less than or equal to 10 cm from anal verge, based on previous conventions in the literature, unless otherwise explained in the study. The primary outcome measured was anastomotic leak.

## Data extraction and quality assessment

Two independent investigators initially screened titles and abstracts for studies to be included, followed by full text reviews of eligible studies. Data extracted include study characteristics, ICG technique, surgical indication, operative and anastomotic details, as well as anastomotic leak. Risk of bias assessment was performed using ROB2, or ROBINS-I risk tool for non-randomised studies.

### Statistical analysis

Network meta-analysis was performed using Bayesian Markov chain Monte Carlo method in WinBUGS^®^ 1.4.3 (MRC Biostatistics Unit, Cambridge, UK), through conduit of Microsoft Excel^®^ (Microsoft, Redmond, WA, USA) based macro NetMetaXL^®^ 1.6.1 (Canadian Agency for Drugs and Technologies in Health). A convergence test was undertaken for each analysis by checking whether the Monte Carlo error was below 5% of the standard deviation of the effect estimates or the variance between the studies. Convergence was achieved for all analyses at 20 000 “burn in” runs and 30 000 model runs. Informative priors with a random-effects model were used to minimise the consequences of the variability of the patient populations and designs for each study.

Clinical postoperative outcomes including anastomotic leak were calculated as odds ratios (OR) with 95 per cent confidence intervals (CI) of direct comparisons between patients with and without ICG at different anastomotic heights.

Plots and tables were generated by the gemtc package in Stata^®^ MP version 15 (StataCorp, College Station, TX, USA). Pairwise comparison tables and graphical rankograms were generated using NetMetaXL^®^. The relative efficacy of anastomotic height and ICG in the prevention of anastomotic leak was also presented as surface under the cumulative ranking (SUCRA) value, ranging from 0 to 1 with 0 being highest chance of being ranked lowest and 1 being highest chance of being ranked best.

Direct and indirect evidence for ICG vs. no ICG at varying anastomotic heights were combined to estimate the examined outcomes, with a 95% equal tail credible interval (CrI). Bayesian analysis was utilised because of its ability to compare multiple groups. We used NetMetaXL in order for rank probabilities to be plotted against the possible ranks for a treatment to result in the production of a graphical “rankogram”.

All results were presented as relative effects and Bayesian estimates of the probability of each technique being the best to the worst relating to every studied outcome with rankograms, league tables and forest plots.

## Results

**Fig. 1:**
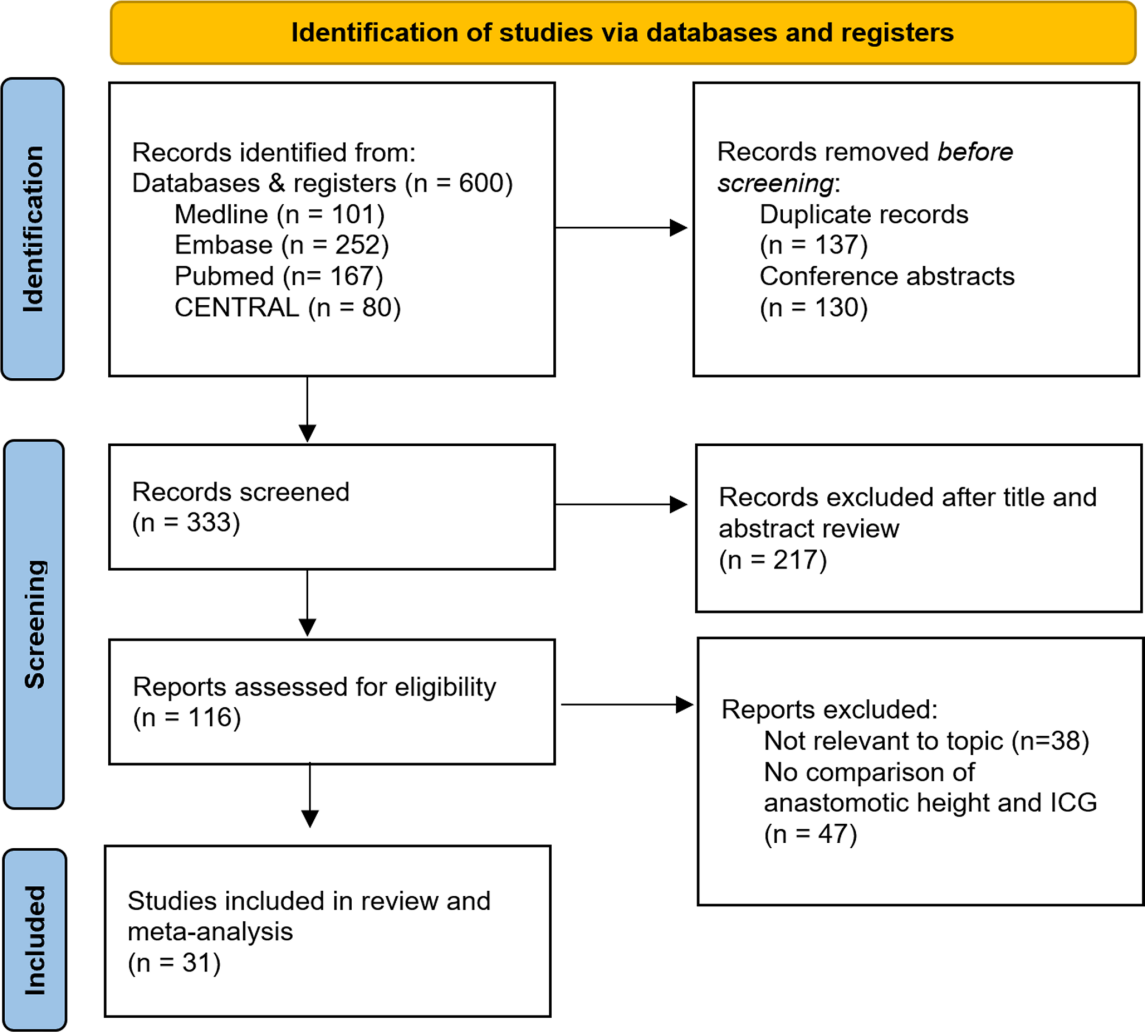
PRISMA diagram of systematic review conducted

Initial database search identified 600 records, of which 333 were identified for screening after removing duplicates and conference abstracts without accompanying journal articles. After title and abstract screen 217 records were excluded due to case reports, reviews or unrelated to the topic of interest. 116 were retrieved for full text review, of which 78 were found to be relevant clinical colorectal studies on the use of ICG, however only 31 met inclusion criteria for network meta-analysis of high and low anastomoses and use of ICG in anastomotic leak rates (Fig. 1). Those studies that compared anastomotic height or use of ICG, without a breakdown of anastomotic leak rates were excluded. Authors of RCTs were contacted for further information if this data was not reported in their published study.

### Characteristics of studies

The 31 studies are listed in Table 1. These included retrospective and prospective cohort studies, with 2 RCTs. Study size ranged from 20 to 1677 patients, and 20 studies included cancer resections only, with the remainder being a combination of benign and malignant indications for resection and anastomosis. The dose and technique of ICG administration, whether before or after anastomosis or both, were noted to be varied. The majority of perfusion assessments were performed laparoscopically, although open and robotic assessments were also described.

**Table 1:** Studies of indocyanine green (ICG) in perfusion assessment of left sided colorectal anastomoses. PSM = Propensity score matching, N.R. = not recorded, n/a = not applicable, al = anastomotic leak, lap = laparoscopic

Study	Year	Enrolment year (country)	Study design	Patients	Population	Intervention and timing relating to anastomosis	Comparison	Outcome	Operative approach (ICG visualisation)	Cancer/Benign
Sherwinter et al. [9]	2013	2010-n.r. (USA)	Prospective cohort	20	Low anterior resection for benign and malignant disease	ICG 2.5 mg after, transanal view	N/A	AL, diversion	Lap (mucosa)	Both
Jafari et al. [10]	2013	2011–2012 (USA)	Retrospective case control	38	Robot assisted LAR	ICG 6–8 mg before	No ICG	AL, revision	Robot assisted (serosa)	Cancer
Ris et al. [11]	2014	2011 (UK)	Prospective cohort	30	Consecutive patients minimally invasive colorectal resection	ICG 2.5 mg after	N/A	AL	Lap (serosa)	Both
Hellan et al. [12]	2014	2012–2013 (USA and Italy)	Prospective cohort	40	Robotic left colon or rectal resection	ICG 10 mg before	N/A	AL, revision	Robot assisted (serosa)	Both
Jafari et al. [13]	2015	2012–2013 (USA)	Prospective cohort	139	Laparoscopic left colectomy or anterior resection	ICG 3.75–7.5 mg before and after	N/A	Feasibility, AL	Lap and robotic assisted (serosa and transanal)	Both
Watanabe et al. [14]	2015	2013–2014 (Japan)	Prospective cohort	119	Consecutive patients left colon or rectal cancer	ICG 0.5 mg/kg before	N/A	Blood flow, AL	Lap and open surgery	Cancer
Protyniak et al. [15]	2015	2013–2014 (USA)	Prospective cohort	77	Laparoscopic colorectal operations	ICG dose N.R., before	N/A	AL	Lap (serosa)	Both
Boni et al. [16]	2016	2013–2014 (Italy)	Prospective cohort	107	Laparoscopic colorectal resections	ICG 0.2 mg/kg before and after	N/A	AL	Lap (serosa)	Both
Boni et al. [17]	2017	2014–2015 (Italy)	Prospective cohort with historical control	80	Laparoscopic anterior resection for cancer	ICG 5 cc of 0.2 mg/kg before	No ICG in historical controls	AL, postoperative complications	Lap (serosa)	Cancer
Kim et al. [18]	2017	2010–2016 (Korea)	Retrospective case cohort, historical control	657	Consecutive robot assisted rectal cancer resections	ICG 10 mg before	No ICG in historical controls	Quantify ICG timing and intensity, AL	Robot (serosa)	Cancer
Wada et al. [19]	2017	2013–2016 (Japan)	Retrospective cohort	112	Laparoscopic surgery for left sided colorectal cancers	ICG 5 mg before	N/A	AL, postoperative outcomes	Lap (serosa)	Cancer
Kawada et al. [20]	2017	2013–2014 (Japan)	Prospective cohort	68	Left sided colorectal cancers for laparosopic resection	ICG 5 mg before	N/A	AL	Lap (serosa)	Cancer
Mizrahi et al. [21]	2018	2013–2016 (USA)	Retrospective cohort, historical control	60	Laparoscopic LAR for cancer	ICG 8.75 mg before and after	No ICG in historical controls	AL, change of surgical plan	Lap (serosa, transanal)	Cancer
Ris et al. [22]	2018	2013–2016 (UK)	Prospective cohort	1677	Elective colorectal patients with anastomoses	ICG 7.5 mg before and after	No ICG in historical controls	AL	Lap, open	Both
Ogino et al. [23]	2019	2017–2018 (Japan)	Prospective cohort	74	Consecutive patients undergoing colorectal surgery	ICG 5 mg before	N/A	AL, revision	Lap, open	Both
Son et al. [24]	2019	2015–2017 (Korea)	Prospective cohort	86	Laparoscopic colorectal surgery for cancer	ICG 0.25 mg/kg before	N/A	AL, anastomotic complication	Lap (serosa)	Cancer
Shapera et al. [25]	2019	2012–2018 (USA)	Retrospective case control	103	Robotic left colon or rectal resection	ICG 25 mg before and after	No ICG in historical controls	AL, anastomotic complication, revision	Robot (serosa)	Both
Wada et al. [26]	2019	2009–2016 (Japan)	Retrospective with PSM	149	Patients undergoing laparoscopic low anterior resection	ICG 5 mg before	No ICG in matched controls	AL	Lap (serosa)	Cancer
Alekseev et al. [27]	2020	2018–2019 (Russia) ‘FLAG’	Randomised Control Trial	377	Sigmoid and rectal resections	ICG 0.2 mg/kg before	No ICG	AL, revision, complications	Lap (serosa)	Cancer
Bonadio et al. [28]	2020	2015–2017 (Italy)	Retrospective cohort	66	Elective laparoscopic anterior resections	ICG 0.2 mg/kg before and after	No ICG in historical controls	AL	Lap (serosa)	Cancer
Foo et al. [29]	2020	2013–2018 (China)	Retrospective cohort	506	Consecutive anterior or low anterior resections	ICG 5–7.5 mg before	No ICG in historical controls, matched	AL, stricture	Open/lap/robot (serosa)	Both
Hasegawa et al. [30]	2020	2007–2017 (Japan)	Retrospective cohort	852	Low anterior resection or intersphincteric resections for rectal cancer	ICG 5 mg before	No ICG in matched controls during the same time period	AL	Lap (serosa)	Cancer
Impellizzeri et al. [31]	2020	2014–2019 (Italy)	Retrospective cohort	196	Patients undergoing elective left sided colorectal surgery	ICG 12.5 mg before and after	No ICG in matched controls during the same time period	AL, revision	Lap (serosa)	Both
Iwamoto et al. [32]	2020	2016–2017 (Japan)	Prospective cohort	25	Laparoscopic anterior resection for rectal cancer	ICG 7.5 mg before	N/A	AL	Lap (serosa)	Cancer
Watanabe et al. [33]	2020	2014–2017 (Japan)	Retrospective cohort	550	Laparoscopic low anterior resection for rectal cancer	ICG 0.25 mg/kg before	No ICG in historical controls, matched	AL	Lap (serosa)	Cancer
Benčurik et al. [34]	2021	2015–2019 (Czech republic)	Prospective cohort with historical control	200	Rectal cancers	ICG 0.2 mg/kg before	No ICG in historical controls	AL	Lap, robotic (serosa)	Cancer
Jafari et al. [6]	2021	2015–2017 (USA) ‘PILLAR III’	Randomised control trial	347	Patients undergoing resection < 10 cm from anal verge	ICG 7.5–10 mg before and after	No ICG	AL	Open, lap, robot (serosa, transanal)	Cancer
Otero-Piñeiro et al. [35]	2021	2011–2018 (Spain)	Retrospective analysis Prospective data	284	TaTME patients	ICG 2.5 mg/mL ‘bolus’ before and after	No ICG in historical controls	AL	Lap (serosa, transanal)	Cancer
Han et al. [36]	2022	2020 (Korea)	Prospective cohort	22	Surgery for rectosigmoid or rectal cancer	High ligation with ICG 7.5 mg before, during and after	Low ligation IMA with ICG	AL	Lap (serosa)	Cancer
Hasegawa et al. [37]	2022	2010–2017 (Japan)	Prospective cohort with historical control	263	Laparoscopic intersphincteric resections	ICG 5 mg before	No ICG in historical controls, matched	Structural sequalae of AL, AL	Lap (serosa)	Cancer
Hagiwara et al. [38]	2023	2018–2022 (Japan)	Retrospective cohort	217	Left sided colon and rectal surgery for cancer	ICG 12.5 mg before	N/A	Time to ICG fluorescence and AL	Open, lap	Cancer

The method of recording high and low anterior resections is outlined in Table 2. Most studies recorded the name of the operation performed, three classified operations by height of anastomoses only. Interestingly, Alekseev et al. [27] and Watanabe [33] both studied LAR, but in their description used cut-offs of anastomoses 8 cm and 12.5 cm from the anal verge respectively. All included studies reported data on HAR and LAR, use of ICG, and subsequent leak rate. All studies recorded symptomatic anastomotic leaks with confirmation; six studies also included asymptomatic anastomotic leaks and those that did not require intervention [27–29, 31, 32, 34]. Four studies included left colectomies with HAR data, they were unable to be separated but had documented leak rates in comparison to the LAR group, and were included [12, 14, 16, 31]. Two RCTs included data for anterior resection, however were not able to provide a breakdown of anastomotic leak rates between HAR and LAR groups, with and without ICG, despite email request, and were unable to be included in this study.

**Table 2:** Studies of indocyanine green (ICG) in perfusion assessment of left sided colorectal anastomoses, har = high anterior resection, lar = low anterior resection, al = anastomotic leak, ICG = indocyanine green, pts = patients

Study	How HAR/LAR Recorded	HAR + ICG		HAR - ICG		LAR + ICG		LAR - ICG	
		AL	Total pts	AL	Total pts	AL	Total pts	AL	Total pts
Sherwinter et al. [9]	By height; HAR > 10 cm, LAR < = 10 cm	1	11			1	9		
Jafari et al. [10]	Operation recorded					1	16	4	22
Ris et al. [11]	Operation recorded	0	18			0	6		
Hellan et al. [12]	Operation recorded, HAR includes left colectomies	0	13			2	27		
Jafari et al. [13]	By height; HAR > = 10 cm, LAR < 10 cm	1	87			1	52		
Watanabe et al. [14]	Operation recorded, HAR includes left colectomies	4	63			3	46		
Protyniak et al. [15]	Operation recorded	0	1			0	47		
Boni et al. [16]	Operation recorded, HAR includes left colectomies, LAR includes ‘>8 cm’	0	35			0	22		
Boni et al. [17]	Operation recorded					0	42	2	38
Kim et al. [18]	Operation recorded					2	310	18	347
Wada et al. [19]	Operation recorded	0	13			5	52		
Hasegawa et al. [20]	Operation recorded	0	10			3	28		
Mizrahi et al. [21]	Operation recorded					0	30	2	30
Ris et al. [22]	Operation recorded	4	191	21	506	3	90	39	365
Ogino et al. [23]	Operation recorded	0	4			1	13		
Son et al. [24]	Operation recorded	0	31			3	55		
Shapera and Hsiung [25]	Operation recorded					0	58	1	23
Wada et al. [26]	Operation recorded					5	48	7	101
Alekseev et al. [27]	By height; HAR = 9–15 cm, LAR < = 8 cm	1	76	4	86	16	111	27	104
Bonadio et al. [28]	LAR = ‘Rectal anterior resection’					2	33	7	33
Foo et al. [29]	HAR = ‘Non-TME’, LAR = ‘TME’ anterior resection	4	126	5	115	6	127	16	138
Hasegawa et al. [30]	Operation recorded					4	141	87	703
Impellizzeri et al. [31]	Operation recorded, HAR includes left colectomies	0	30	4	62	0	37	2	36
Iwamoto et al. [32]	Operation recorded	1	6			5	19		
Watanabe et al. [33]	Only LAR cases, but included anastomoses up to 12.5 cm					10	236	22	314
Benčurik et al. [34]	Operation recorded					9	100	19	100
Jafari et al. [6]	Operation recorded					16	178	16	169
Otero-Piñeiro et al. [35]	Operation recorded	0	18			2	62		
Han et al. [36]	Operation recorded	0	10			2	12		
Hasegawa et al. [37]	Operation recorded					4	70	31	193
Hagiwara et al. [38]	Operation recorded	1	45			15	81		

## Risk of bias assessment

**Fig. 2:**
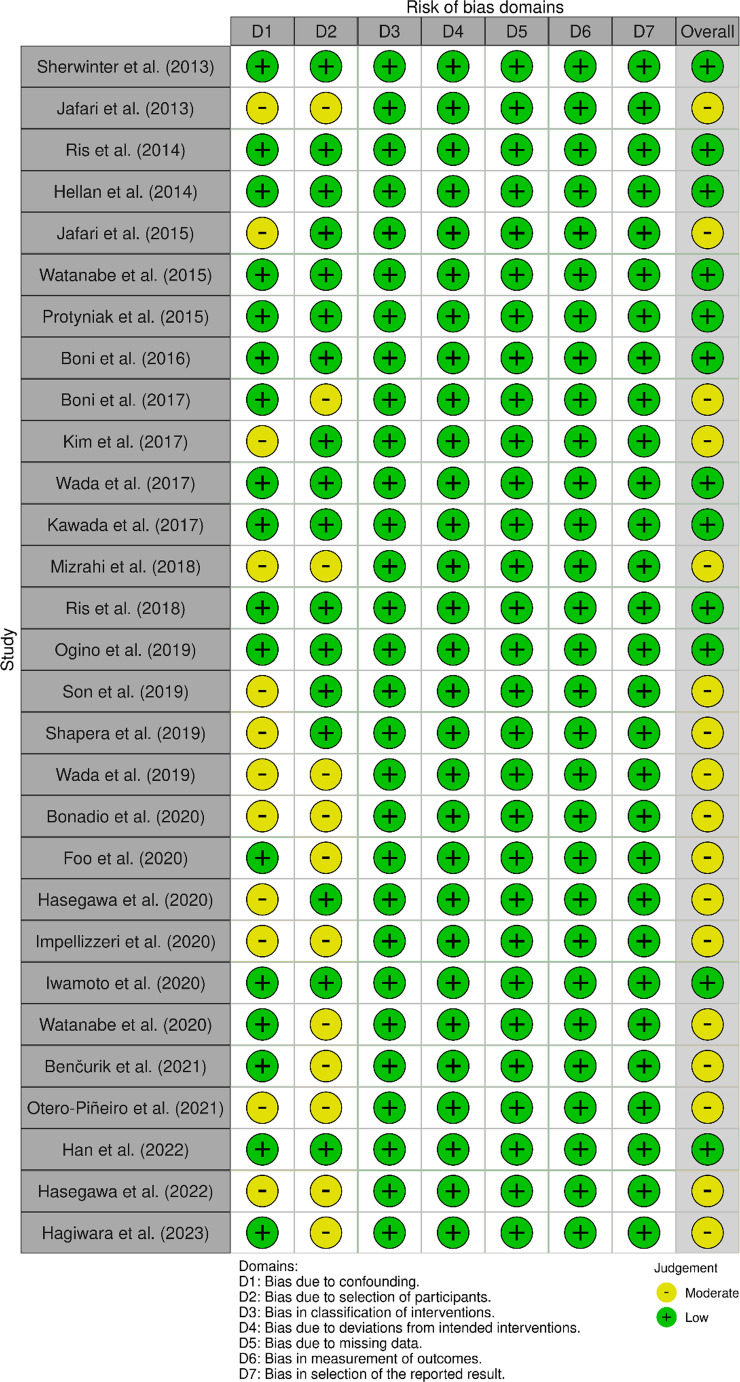

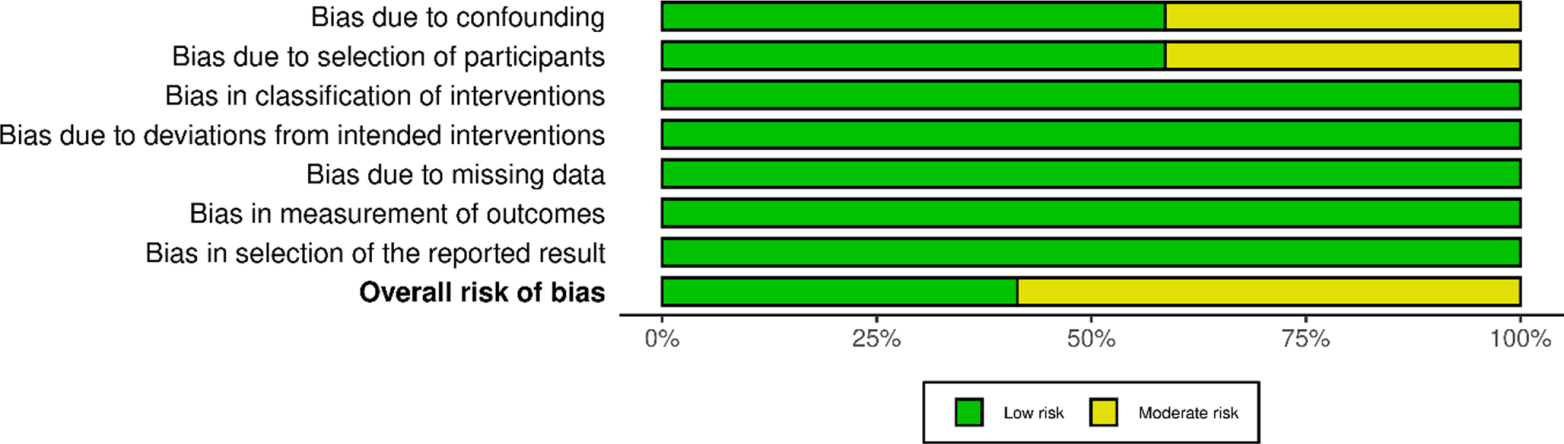
Risk of Bias in non-randomised studies using ROBINS-I tool

**Fig. 3:**
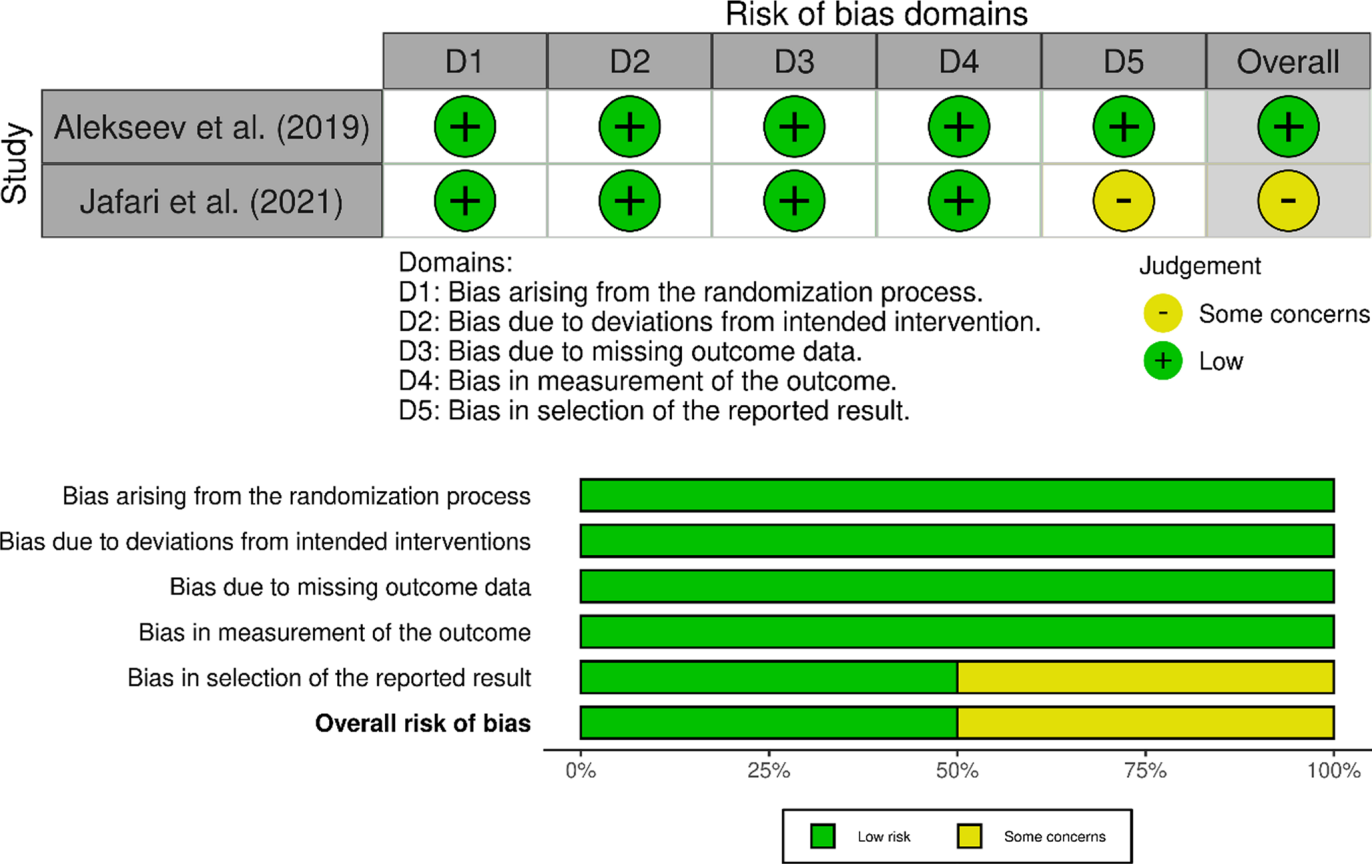
Risk of bias in randomised studies using ROB2 tool

Risk of Bias was assessed using ROBINS-I for non-randomised studies and ROB2 for RCTs. Results are shown in Figs. 2 and 3. Non-randomised studies were low to moderate risk mostly due to risk of confounding or selection from historical controls, although propensity score matching were often used to adjust for this.

## Network meta-analysis

**Fig. 4:**
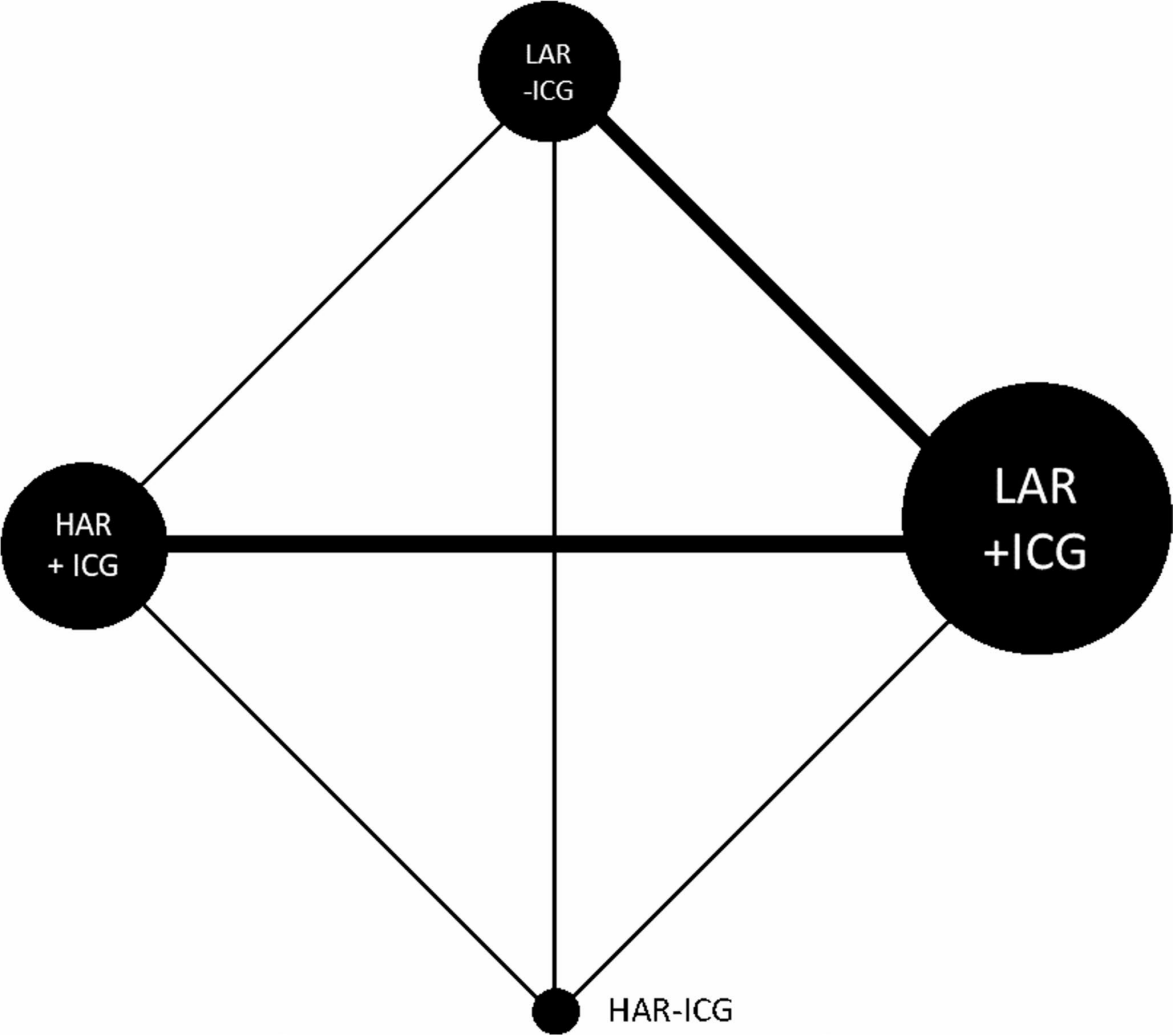
Network plot for direct comparisons of anastomotic leak in studies of High anterior resection (HAR) and Low anterior resection (LAR) and use of indocyanine green (ICG)

There were 31 studies, with 6431 patients, included in the analysis. Details of the network characteristics and events can be found in Supplementary Information S2. Direct comparisons between HAR, LAR, with and without ICG, ranged from 4 to 18 studies, with 1066 to 4343 patients compared. Figure 4 shows a network plot of these studies.

**Fig. 5:**
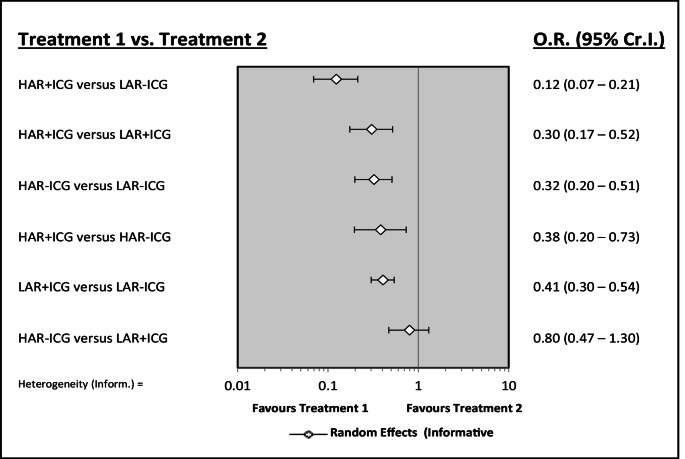
Forest plot comparing interventions in relation to rates of anastomotic leak in studies of High anterior resection (HAR) and Low anterior resection (LAR) and use of indocyanine green (ICG)

**Fig. 6:**
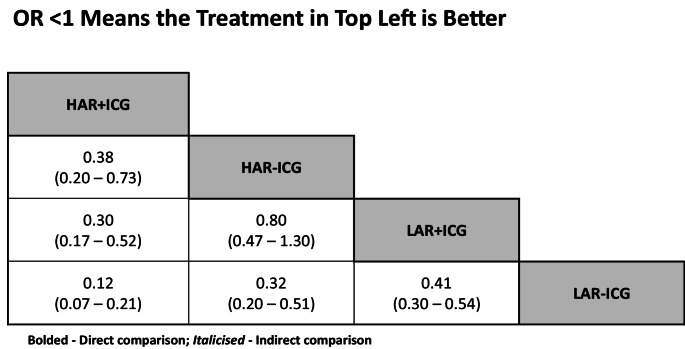
Pairwise comparisons of odds ratios of anastomotic leak, in network meta-analysis of studies of High anterior resection (HAR) and Low anterior resection (LAR) and with and without use of indocyanine green (ICG)

**Fig. 7:**
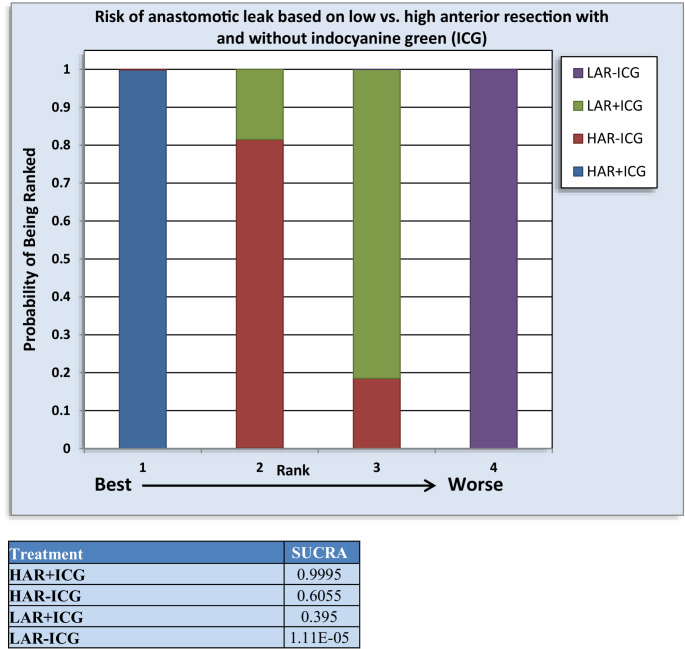
Rankogram with probability for ranking first to fourth (least likely to be associated with anastomotic leak to most likely respectively). The probability ranges from 1.0 (most likely to fit in assigned ranking) to 0 (least likely). Studies are based on low (LAR) vs. high anterior resection (HAR) with and without indocyanine green (ICG). Intervention on left of graph, with higher surface under the cumulative ranking curve (SUCRA) is best

Odds of leak is greater in LAR compared to HAR. With or without ICG, odds of an anastomotic leak is 0.30 (0.17–0.52) and 0.32 (0.20–0.51), favouring HAR over LAR. ICG is protective in both HAR and LAR. Odds of anastomotic leak is 0.38 (0.20–0.73) in HAR with ICG compared to without, and for LAR the odds of leak are 0.41 (0.30–0.54) comparing with ICG to without (Figs. 5 and 6). A rankogram demonstrates HAR with ICG is the best intervention at reducing anastomotic leak, whereas LAR without ICG is the worst (Fig. 7).

## Discussion

Anastomotic leak is a potentially life-threatening complication of colorectal surgery. Perfusion of the anastomosis is a major risk factor that can be assessed using ICG-FA. Lower anastomoses is also associated with increased leak rates when compared to high anastomosis. Despite the difference in leak rates and potential factors contributing to anastomotic leak between the two groups, current meta-analyses have not separately studied the benefit of ICG-FA in high and low anastomoses.

This study demonstrates that in comparing high and low anastomoses with and without ICG, the use of ICG is protective in both HAR and LAR. This network meta-analysis adds to the growing evidence that demonstrate the benefit of ICG in colorectal anastomoses. Early studies were mixed, with Kin et al. [39] reporting that ICG did not decrease the rate of anastomotic leak in their 173 case matched pairs, with two subsequent RCTs by De Nardi et al. [5] and Jafari et al. [6] that did not find a significant decrease in leak rates. However, with more recent data and several larger studies, Safiejko et al. [40] in their systematic review and meta-analysis of 32 studies in colorectal cancer surgery alone, showed significantly lower rates of anastomotic leak using ICG, with pooled results of 3.7% with ICG and 7.6% without (*p* < 0.001). Tang et al. [41] showed ICG reduced the incidence of AL (OR 0.46; 95% CI, 0.36–0.59) without prolonging operating time or affecting rates of post-operative complications in propensity score matched studies and RCTs. Lucarini et al. [42] analysed four RCTs on rectal surgery only, with 1510 patients, finding anastomotic leak rates of 9% and 13.9%, with and without ICG respectively (*p* < 0.003).

The leak rate and factors contributing to anastomotic leak in HAR and LAR may be significantly different. Therefore, assessing the effectiveness of ICG in reducing the risk of leak in left-sided resections without stratifying by height of anastomosis may be a confounder. In many older studies, including RCTs, this distinction is not made, resulting in a heterogenous group of patients and operations. They may be recorded as left-sided colorectal resections, or, studies may include right sided resections also [5, 43]. More recent studies on low and ultralow anterior resections only avoids the confounder of higher joints, and highlights the clinical interest in low anastomoses [37].

Low anastomosis is thought to be associated with higher risk of leak because technical challenges may result in impaired blood supply; there may be difficulties in forming the anastomosis, requiring multiple stapler firings for rectal transection prior to circular stapled anastomosis, compounded by the anatomical constraints of a narrow male pelvis, and there may be the presence of pelvic hematoma, as suggested in several papers on anastomotic leak [8, 44, 45]. Other factors that may contribute to anastomotic leak include tension of anastomoses, technical expertise, microbiome, neoadjuvant treatment, steroids, NSAIDs and distal obstruction. However, these are out of the scope and study aims of this network meta-analysis. Zarnescu et al. [44] in their recent review noted an anastomotic height of less than 7 cm is an independent risk factor for leak, and less than 5 cm can be associated with up to 6.5 times higher risk of leak compared with higher anastomoses. Given the height of the pathology, such as a rectal cancer, is non-modifiable, along with the anatomy of the bony pelvis, attention turns to modifiable factors, in particular the microcirculation. Our study is therefore in keeping with a higher rate of leak seen in low anastomoses, but in particular shows the benefit of ICG to assess anastomotic perfusion in this group, to reduce the rate of leak.

Interestingly, we also found that despite being advantageous in both, ICG was more beneficial in HAR compared to LAR, with a lower OR of leak. The reason for this is unclear. It may be related to more data available for LAR, including studies that found equivocal benefit for the use of ICG-FA, and fewer patients in the HAR group, resulting in a wider confidence interval for HAR, and slightly lower calculated OR. There may be more factors at play in a low anastomosis; patient factors including pre-operative radiotherapy, and technical factors such as tension and the technical challenges in accessing the low, narrow pelvis, such that ischemia only plays a part and the effect of ICG-FA on the rate of anastomotic leak is less pronounced. These other factors are less common in HAR. This is a similar explanation to the finding by Emile et al. [7] of 27 studies that found a change in surgical plan using ICG-FA was actually associated with a higher odds ratio of leak, OR 2.73 (95% CI 1.54–4.82), with the authors suggesting anastomoses requiring revision due to poor perfusion may still remain high risk and susceptible to post-operative changes, or that more proximal transection may introduce tension, or that other factors affecting anastomotic healing remain.

It is recognised that there is a paucity of large randomised controlled trials into the use of ICG-FA in colorectal anastomoses, with most studies being prospective cohort studies. This analysis included all studies identified during electronic search, including recent larger studies and two RCTs, over 6000 patients. RCTs in ICG-FA often need large numbers for adequate study power. The first landmark RCT by De Nardi et al. [5] showed a trend toward ICG-FA reducing rates of anastomotic leak but did not reach significance, and the PILLAR III industry-funded RCT was terminated early due to slow recruitment [6]. Most recently, the results of the EssentiAL study was published, with 839 patients across 41 centers, and showed a significant reduction in anastomotic leak with ICG, in minimally invasive sphincter preserving surgery for rectal cancer [46]. The European IntACT and Dutch AVOID RCTs both planned for large numbers for recruitment; 880 in IntACT in order to demonstrate a leak rate reduction of 12–6%, and 978 in AVOID for a reduction from 7 to 3%, and were ongoing at the time of this review [47].

There were limitations to this study. One is the known considerable variation in ICG administration, reflecting a lack of consensus on optimal timing and dose in ICG-FA, limiting the generalisability of findings. A recent Delphi study of 35 international experts by Wexner et al. [48] recommended 5–10 mg of ICG, 30–60 s before assessment, dosing by weight, and re-administration if necessary, based on 70% consensus among them. Other systematic reviews have noted the heterogeneity in reported techniques, although concluded that all appear to be within safe dosing [7, 49]. Additionally, there is interest in obtaining a more objective measurement of fluorescence. Studies have shown a wide inter-observer variation when assessing fluorescence visually [50, 51]. Visual grading systems and quantitative measurements using software derived fluorescence curves have been described with a view to overcome this [4, 14, 24, 52]. However, differences in technique and software, and the presence of variables such as distance to the tissue of interest and ICG dosing, have meant that there is yet to be a widely accepted quantitative method [53].

There were also differences in the definition of HAR and LAR used by some studies. The majority of studies listed the operation, but only some mentioned that their definition is based on the relationship of the anastomosis to the peritoneal reflection [11, 22]. A minority used measurements. Some were clear in their definition of this, for example, Jafari et al. [6] stated all LAR were defined as anastomoses within 10 cm from the anal verge. These definitions and conventions likely persisted from a time when rectal cancer diagnosis and treatment were based on the endoscopic distance from the anus. The Dutch TME study for example classified rectal cancers based on distance < = 5 cm, 5–10 cm, or 10–15 cm from the anal verge, and reported their responses to radiotherapy respectively [54]. Previously, rigid proctoscopy or sigmoidoscopy were common assessments of rectal tumours and anastomoses. Nowadays, MRI is more frequently used to define rectal cancers, based on radiological landmarks. However, the terms HAR and LAR continue to be used, and may include benign and malignant conditions, and sigmoid as well as rectal pathologies. When the anastomosis is less than 10 cm from the anal verge, it is still considered a LAR, and above this to be a HAR, in literature on anastomoses and more recently, low anterior resection syndrome [55, 56]. From our perspective, we were interested in the clinical application in left sided resections, and selected the studies based on the above definitions of HAR and LAR. Interestingly, some listed LAR as the operation used, but the anastomotic height was noted to be 8–12.5 cm in the description [27, 33]. These were taken to mean LAR as the studies intended, as no further breakdown was available on review of the study data, and still showed that LAR had greater odds of anastomotic leak. Bonadio [28] and Foo [29] used unconventional terms such as rectal anterior resection and TME anterior resection, however examination of the study methodology correlated these with LAR. This demonstrates the inconsistencies that still exist in reporting.

Likewise, there is still variability in the reporting of anastomotic leak. The majority of the included studies reported on symptomatic anastomotic leak only, at 30 days, with investigation to confirm diagnosis based on clinical suspicion. Some, though not all, used the grading system developed by the International Study Group on Rectal Cancer, Grade A-C, based on the intervention required [57]. Several studies specifically mentioned that routine pelvic CT or contrast enemas were not performed at their institution, whereas other studies such as the FLAG trial routinely screened for radiological leaks by 30 days [19, 20, 27, 38]. There is therefore possibility of missed Grade A, asymptomatic leaks. Studies rarely extended beyond 30-day follow-up and therefore, delayed anastomotic complications may also be missed.

Similarly, some studies combined left colectomies, sigmoid colectomies and HAR [12, 14, 16, 31]. These studies were only included if there was documented anastomotic leak rates in comparison to the LAR group, and excluded otherwise. Often studies compared left sided resections with data on LAR but did not break down the other group to the same detail. Of note, two RCTs were identified during screening that included left sided resections but did not have data published on leak rates in association with the height of anastomosis and use of ICG. Authors were contacted via email however did not supply the requested data, one due to ongoing subgroup analysis. These studies ultimately had to be excluded from this network meta-analysis. Despite the above limitations, this network meta-analysis demonstrated that ICG had benefit in reducing anastomotic leak in both HAR and LAR.

## Conclusion

The utilisation of ICG to check for perfusion is beneficial and protective in terms of anastomotic leak in both HAR and LAR. While there is no consensus on the application, dosage and timing of ICG during anterior resection, pooled results and most studies have shown that the use of ICG to check for tissue perfusion of anastomosis reduces leak. ICG should therefore be considered as part of a surgeon’s usual armamentarium for intraoperative anastomotic check to reduce the risk of postoperative anastomotic leak.

## Electronic supplementary material

Below is the link to the electronic supplementary material.

Supplementary Material 1

## Data Availability

Data is provided within the manuscript or supplementary information files.
