# Supplementary material for: Indocyanine green and height of anastomosis in colorectal surgery– a network meta-analysis
Source: Langenbecks Arch Surg. 2025 Jun 12;410(1):187. doi: 10.1007/s00423-025-03765-x (PMC12159106; doi:10.1007/s00423-025-03765-x)
Supplement: Supplementary file 1 — Supplementary Material 1 [file 423_2025_3765_MOESM1_ESM.pdf]

Supplementary Information

Article Title: Indocyanine Green and height of anastomosis in colorectal surgery – a network meta-analysis

Journal Name: Langenbeck's Archives of Surgery

Author: Fok, Kar Yin[1,2], Toh, James Wei-Tatt[1,2]

Corresponding author:

Fok, Kar Yin

[1]Westmead Hospital, Cnr Hawkesbury and Darcy Rds, Westmead, NSW 2047, Australia

Email: kyfok01@gmail.com

[2] University of Sydney, Camperdown NSW 2050, Australia

S1: Medline search strategy

MEDLINE(R) including Daily update <1996-current>

1	exp Indocyanine Green/	8217
2	Colorectal Surgery/	3586
3	exp Fluorescence/	34423
4	exp Anastomosis, Surgical/	73371
5	exp Perfusion/	29194
6	exp Anastomotic Leak/	5138
7	exp Postoperative Complications/	427564
8	exp Perfusion Imaging/	8813
9	ICG*.mp.	8260
10	ICG-FA.mp.	55
11	exp colon/ or exp rectum/	62507
12	2 or 11	65837
13	1 or 3 or 5 or 8 or 9 or 10	82701
14	6 or 7	427564
15	12 and 13 and 14	100
16	limit 15 to humans	98
17	limit 16 to english language	89

***S1:** Example of electronic search strategy via OVID Medline*

S2: Network Characteristics

Network Characteristics

Characteristic	Number
Number of Interventions	4
Number of Studies	31
Total Number of Patients in Network	0
Total Number of Events in Network	472
Total Possible Pairwise Comparisons	6
Total Number Pairwise Comparisons With Direct Data	6
Number of Two-arm Studies	27
Number of Multi-Arms Studies	4
Number of Studies With No Zero Events	17
Number of Studies With At Least One Zero Event	14
Number of Studies with All Zero Events	3

Intervention Characteristics

Treatment	# Studies	# Events	# Patients	Aggregate Rate	Min. Rate	Max. Rate
HAR+ICG	19	17	788	0.0216	0.0115	0.1667
HAR-ICG	4	34	769	0.0442	0.0415	0.0645
LAR+ICG	31	121	2,158	0.0561	0.0065	0.2632
LAR-ICG	16	300	2716	0.1105	0.0435	0.2596

Direct Comparison Characteristics

Comparison	# Studies	# Patients	# Events
HAR+ICG vs. LAR+ICG	18	1,684	85
LAR+ICG vs. LAR-ICG	16	4,343	378
HAR+ICG vs. HAR-ICG	4	1,192	43
HAR+ICG vs. LAR-ICG	4	1,066	93
HAR-ICG vs. LAR+ICG	4	1,134	59
HAR-ICG vs. LAR-ICG	4	1,412	118

S2: *Network characteristics of the network meta-analysis performed on studies comparing anastomotic leak in studies of High anterior resection (HAR) and Low anterior resection (LAR) with and without (+/-) the use of indocyanine green (ICG)*
